# Quality of life of Lithuanian women with early stage breast cancer

**DOI:** 10.1186/1471-2458-7-124

**Published:** 2007-06-26

**Authors:** Giedre Bulotiene, Jonas Veseliunas, Valerijus Ostapenko

**Affiliations:** 1 Department of Physical Medicine and Rehabilitation, Institute of Oncology, Vilnius University, Vilnius, Lithuania; 2 Department of Breast Surgery and Oncology, Institute of Oncology, Vilnius University, Vilnius, Lithuania

## Abstract

**Background:**

In the last decades, there have been no studies carried out in Lithuania on the quality of life of breast cancer patients. The aim of the present study was to evaluate changes in the quality of life of Lithuanian women with the early stage of breast cancer nine months after surgery and its dependence on surgical strategy, adjuvant chemotherapy and the social and demographic status of the patients.

**Methods:**

Seventy-seven patients with early stage breast cancer filled in the FACT-An questionnaire twice: one week and nine months after the surgery. The main age of the patients was 53.1 ± 10.6 years. We distinguished the mastectomy group and breast conserving treatment (BCT) group with/without chemotherapy. The groups were identical in their social and demographic status (age, education, occupation and marital status). Changes in the quality of life in these groups were compared nine months after surgery.

**Results:**

Nine months after surgery, the overall quality of life was found worse in both mastectomy and BCT groups. Changes were induced by the worsening of the emotional and social well-being. The quality of life became worse in the mastectomy plus chemotherapy sample. No changes were detected in the mastectomy group without chemotherapy. In addition, the multivariate analysis showed that the marital status was quite a significant determinant of the functional well-being.

**Conclusion:**

Nine months after surgery, the study revealed a worsening of the overall quality of life in both groups of patients – those who had undergone mastectomy and BCT. The quality of life became considerably worse in the mastectomy plus chemotherapy group. Marital status was found to exert the most considerable influence on the women's quality of life in comparison with other social and demographic factors.

## Background

Numerous studies on the quality of life of breast cancer patients, carried out in the last decades, presented rather scarce data on the psychosocial aspects of the transitional period between the primary treatment and survival [1]. The first year is most stressful because of the adaptation to the impact of the diagnosis, treatment and anxiety caused by the follow-up visits and tests [2].

A review of the literature [3] has shown that the women's psychological vulnerability and adaptation after mastectomy and breast conserving treatment (BCT) differ insignificantly, except the body image, which is better after BCT. A later 5-year prospective study [4] has shown that although some quality of life domains became better after BCT than after mastectomy, there was a direct relation to the patients' age and other sociodemographic factors.

Studies have pointed out the importance of chemotherapy for the quality of life after the surgical treatment of breast cancer [1,5]. Chemotherapy in breast cancer 0-II stages lowered the quality of life scores overall across the four dimensions with a disproportionately greater impact on women with lower level of education [5].

Nausea and vomiting are common side effects of adjuvant chemotherapy. Additional troublesome effects are hair loss, weight gain and concentration problems. A disruptive effect of chemotherapy on younger women is premature menopause. One more distressing symptom that can last particularly long after the treatment is fatigue [6]. The Anemia Subscale of the Functional Assessment of Cancer Therapy (FACT-An) is able to capture the physical and psychological aspects of fatigue [7].

The aim of our study was to evaluate changes in the quality of life of Lithuanian women with the early stage of breast cancer nine months following surgery and its dependence on the surgical strategy, adjuvant chemotherapy, social and demographic status.

## Methods

This study is part of an extended prospective longitudinal study on the early stage breast cancer patients. It was carried out at the Department of Breast Surgery and Oncology of the Institute of Oncology, Vilnius University in the period between January 2004 and December 2005. 117 patients with T1-T2/N0-N1/M0 stages of breast cancer were enrolled in the study. All women signed an informed consent form approved by the Lithuanian Bioethics Committee. Data on the patients' diagnosis, treatment and age were taken from the patients' records. The Functional Assessment of Cancer Therapy General questionnaire (FACT-G) along with the additional Anemia module (FACT-An) was completed a week after surgery. On-treatment assessment was necessary to compare the results [8]. The permission to use FACT-An for this study was received from the authors. FACT-An was sent to the patients by mail nine months later. Responses were received from 77 (66%) patients. The patients who did not send written answers were contacted by phone to find out the reasons. The most frequent explanation was: "because I felt bad" or "I didn't understand some questions"; the other reasons were: "at that time I was away" or "I didn't get the mail". Only a few were traced to have changed their address or phone number. Seventy-seven patients who completed the questionnaires twice were included into the study.

The FACT-G is a 27-item compilation of general questions divided into four primary QOL domains: Physical well-being (PWB), Social/Family well-being (SWB), Emotional well-being (EWB) and Functional well-being (FWB). Each domain comprises six to seven questions scored by the use of a Likert-type scale ranging from 0 (not at all) to 4 (very much). The Total FACT-G score is obtained by summing up four individual subscale scores (PWB, EWB, SWB and FWB). The FACT-An includes 13 items that attempt to identify the intensity of fatigue experienced during the 7-day period before the questionnaire administration, plus seven additional items (20 in total) pertaining to symptoms associated with anemic processes [7]. The Total FACT-An score is a sum of four subscales plus the Anemia subscale. The Trial Outcome Index (TOI) is a sum of PWB, FWB and Anemia subscales. The TOI is an efficient summary index of physical/functional outcomes. All scales are scored so that a higher score shows the better quality of life.

The patients were divided into groups according to their treatment and sociodemographic characteristics. Changes in the quality of life in different groups nine months following surgery were compared.

The t-test for dependent samples was used to compare the main score values for the QOL domains. The significance was set at p < 0.05. Multivariate analysis was performed to identify predictors for a poor QOL during the follow up. The statistical program for FACIT scoring together with SAS was used for data processing.

## Results

The mean age of patients was 53.1 ± 10.6 years (range 32 – 78). The patients' distribution according to their treatment and sociodemographic characteristics is presented in Table 1.

**Table 1:** Clinical and demographic characteristics of patients

Variable	Group	N	N (%)
Total	Mastectomy	30	39
	BCT	47	61
Mastectomy	without chemo	14	18
	with chemo	16	21
BCT	without chemo	21	27
	with chemo	26	34
Education	College or less	38	49
	University	39	51
Occupation	Employed	51	66
	Not employed	26	34
Marital status	Married	43	56
	Not married (unmarried, widowed or divorced)	34	44

Nine months after surgery, the overall quality of life (Total FACT-G) scores and Total FACT-An scores showed a decrease in both mastectomy and BCT groups (Table 2). The social well-being (SWB) scores showed a decrease in the mastectomy group, and the social well-being and emotional well-being (EWB) scores became lower in the BCT group.

**Table 2:** FACT scores that significantly changed over time

	One week after surgery (mean ± SD)	9 months after surgery (mean ± SD)	p value
Total (n = 77)			
SWB	21.5 ± 4.7	17.2 ± 5.9	0.000 000
EWB	17.5 ± 4.9	16.1 ± 5.3	0.0055
FACT-Anemia	61.4 ± 11.2	58.4 ± 13.9	0.019
Total FACT-G	79.2 ± 13.3	72.9 ± 16.9	0.0006
Total FACT-An	140.6 ± 22.2	131.4 ± 28.8	0.00099
Mastectomy (n = 30)			
SWB	22.4 ± 4.5	17.5 ± 5.9	0.00018
Total FACT-G	80.5 ± 13.8	73.7 ± 16.7	0.033
Total FACT-An	143.2 ± 22.9	132.7 ± 28.5	0.041
BCT (n = 47)			
SWB	21.0 ± 4.7	17.1 ± 6.0	0.000054
EWB	17.9 ± 4.8	15.7 ± 5.2	0.0017
Total FACT-G	78.3 ± 13.1	72.6 ± 17.2	0.007
Total FACT-An	138.9 ± 21.8	130.5 ± 29.2	0.01
Mastectomy with chemo (n = 16)			
SWB	22.6 ± 4.5	16.4 ± 6.0	0.0004
EWB	18.9 ± 4.2	16.8 ± 5.1	0.059*
FWB	19.6 ± 4.1	15.5 ± 6.0	0.057*
Total FACT-G	85.1 ± 11.9	71.1 ± 16.2	0.005
Total FACT-An	150.9 ± 18.0	130.8 ± 27.5	0.012
TOI-Anemia	109.4 ± 133.3	97.6 ± 20.5	0.043
BCT with chemo (n = 26)			
SWB	21.2 ± 5.7	16.3 ± 6.9	0.0015
EWB	17.6 ± 4.6	14.8 ± 5.3	0.0046
Total FACT-G	77.8 ± 15.1	69.9 ± 18.2	0.019
Total FACT-An	137.6 ± 24.7	127.1 ± 31.6	0.038
BCT without chemo (n = 21)			
SWB	20.8 ± 3.4	17.9 ± 4.6	0.012

* Close to significant

In the groups of mastectomy with/without chemotherapy and BCT with/without chemotherapy, QOL decreased mostly in the mastectomy plus chemotherapy group (SWB, EWB, FWB, Total FACT-G, Total FACT-An and TOI-Anemia scores show decrease) (Table 2). In the mastectomy group without chemotherapy, the QOL scores remained the same as one week after surgery.

In the BCT with chemotherapy group, SWB, EWB, Total FACT-G and Total FACT-An scores showed a decrease. In the group BCT without chemotherapy, only SWB scores showed a significant decrease (Table 2).

In all the patients, the SWB, EWB, FACT-An, Total FACT-G and Total FACT-An scores decreased significantly over time (Table 2). These findings indicate a worsening of QOL, as a result of the worsening in the emotional and social well-being domains.

Multivariate regressive analysis shows the marital status to be a significant determinant of changes in the functional well-being domain of QOL (Table 3).

**Table 3:** Results of multivariate regressive analysis (p values)

Variable	PWB	SWB	EWB	FWB	FACT-Anemia	Total FACT-G	Total FACT-An	TOI-Anemia
Age	0.95	0.98	0.3	0.69	0.76	0.65	0.88	0.95
Education	0.34	0.16	0.34	0.33	0.1	0.12	0.078	0.11
Occupation	0.91	0.14	0.52	0.11	0.19	0.77	0.68	0.85
Marital status	0.82	0.83	0.22	0.045*	0.19	0.73	0.40	0.15

* Statistically significant

**Figure 1:**
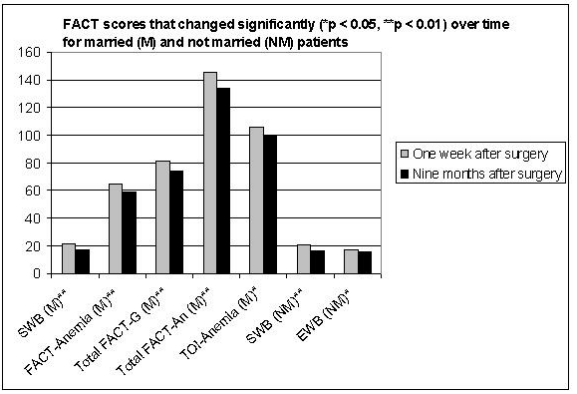
FACT scores that changed significantly (*p < 0.05, **p < 0.01) over time for married (M) and not married (NM) patients.

In the group of married women, the SWB, FACT-An, Total FACT-G, Total FACT-An and TOI-Anemia scores showed a significant decrease nine months after surgery (Figure 1). In the group of unmarried women, only the SWB and EWB scores significantly decreased over time. The respondents' marital status had no significant influence on their participation in the second interview.

## Discussion

Some specific study limitations were the lack of data about QOL of those who did not respond to the questionnaires. Besides, the patients answered the questionnaires for the first time in the hospital and the second time at home, and this might have influenced their answers. The second limitation of the study was a small number of patients, who were divided into four groups: mastectomy with/without chemotherapy and BCT with/without chemotherapy. This might have reduced the possibility of generalizing the results.

The worsening was caused mostly by the lowered social well-being scores in both groups and the lower emotional well-being in the BCT group. No significant changes were detected in the patients' physical well-being nine months after surgery. Although some authors pointed out that the social and emotional well-being shows no rapid or dramatical changes over time or in response to physical health interventions [11], in our study the social and emotional well-being subscales were rather sensitive to reflect changes in the patients' quality of life after surgery and chemotherapy.

One of the possible reasons for the QOL worsening the social domain nine months after surgery might be the changed role of the women in their family after the illness or treatment. Their QOL was significantly influenced also by the marital status, more than by any other social and demographic variables including age, education and occupation (Table 3). These data on the QOL of Lithuanian women with early stage breast cancer differed from the findings of other researchers [4,5].

The declining quality of life in the BCT group in the emotional and social domains confirmed the suggestion that women treated with breast conservation often perceive themselves as receiving less emotional support than those who underwent mastectomy [3,6]. The high quality life in the group of the mastectomy without chemotherapy showed the high adaptation possibilities of these patients.

In our study, statistically significant (p < 0.05) changes in QOL scores corresponded to the minimally important differences (MID) for scores of selected FACT scales and subscales [9-11]: 2 points for EWB [12,13], 2 – 3 points for FWB [12], 3 – 7 points for Total FACT-G [12,14-16], 6 points for TOI-Anemia and 7 points for Total FACT-An [14].

## Conclusion

1. The overall quality of life declined nine months after surgery in both groups of patients – those after mastectomy and after BCT: social well-being worsened in the mastectomy sample, while the social and emotional well-being worsened in the BCT sample.

2. Nine months after surgery, the quality of life (SWB, EWB, FWB, FACT-An TOI, Total FACT-G, Total FACT-An and FACT-An scores) was the worst in mastectomy plus chemotherapy group. No change in the quality of life was found among the patients after mastectomy without chemotherapy: nine months after surgery the quality of life scores remained the same.

3. Marital status, more than the other social and demographic factors, has been found to influence changes in the quality of life of Lithuanian women nine months after surgery.

## Competing interests

The author(s) declare that they have no competing interests.

## Authors' contributions

GB designed the study, participated in data collection and statistical analysis and drafted the manuscript. JV coordinated the study and provided a critical review of the manuscript. VO participated in the design and coordination of the study and contributed to the intellectual content of the manuscript. All the authors have read and approved the final version of the manuscript.

## Pre-publication history

The pre-publication history for this paper can be accessed here:


